# Characterization of Five Transmembrane Proteins: With Focus on the Tweety, Sideroflexin, and YIP1 Domain Families

**DOI:** 10.3389/fcell.2021.708754

**Published:** 2021-07-19

**Authors:** Misty M. Attwood, Helgi B. Schiöth

**Affiliations:** ^1^Functional Pharmacology, Department of Neuroscience, Uppsala University, Uppsala, Sweden; ^2^Institute for Translational Medicine and Biotechnology, Sechenov First Moscow State Medical University, Moscow, Russia

**Keywords:** protein trafficking, tweety family, sideroflexin family, YIPF family, cancer prognostic marker, transmembrane protein

## Abstract

Transmembrane proteins are involved in many essential cell processes such as signal transduction, transport, and protein trafficking, and hence many are implicated in different disease pathways. Further, as the structure and function of proteins are correlated, investigating a group of proteins with the same tertiary structure, i.e., the same number of transmembrane regions, may give understanding about their functional roles and potential as therapeutic targets. This analysis investigates the previously unstudied group of proteins with five transmembrane-spanning regions (5TM). More than half of the 58 proteins identified with the 5TM architecture belong to 12 families with two or more members. Interestingly, more than half the proteins in the dataset function in localization activities through movement or tethering of cell components and more than one-third are involved in transport activities, particularly in the mitochondria. Surprisingly, no receptor activity was identified within this dataset in large contrast with other TM groups. The three major 5TM families, which comprise nearly 30% of the dataset, include the tweety family, the sideroflexin family and the Yip1 domain (YIPF) family. We also analyzed the evolutionary origin of these three families. The YIPF family appears to be the most ancient with presence in bacteria and archaea, while the tweety and sideroflexin families are first found in eukaryotes. We found no evidence of common decent for these three families. About 30% of the 5TM proteins have prominent expression in the brain, liver, or testis. Importantly, 60% of these proteins are identified as cancer prognostic markers, where they are associated with clinical outcomes of various tumor types. Nearly 10% of the 5TMs are still not fully characterized and further investigation of their functional activities and expression is warranted. This study provides the first comprehensive analysis of proteins with the 5TM architecture, providing details of their unique characteristics.

## Introduction

Approximately 25–30% of the ∼20,000 protein coding genes in *Homo sapiens* code for alpha-helical transmembrane proteins (Almén et al., 2009; Fagerberg et al., 2010; Attwood et al., 2017). The ∼5,500 transmembrane proteins have amino (N)- or carboxyl (C)-terminal domains that reside in either the cytoplasmic environment or non-cytoplasmic/lumen/extra-cellular environment and contribute to the functional activities of the protein. Transmembrane (TM) proteins are involved in many crucial cell processes including receptor and signaling transduction pathways, transport of ions and molecules across impermeable membranes, protein targeting and intracellular transport, as well as membrane trafficking (Müller et al., 2008). Additionally, subcellular compartments within cells are maintained by membranes and organelle-specific activities are based on the distribution and function of different transmembrane proteins. For example, vesicle formation and trafficking at the Golgi apparatus, protein targeting and trafficking at the endoplasmic reticulum (ER), or receptor signaling at the plasma membrane. Further, the development of organelles has been aided by the evolutionary retargeting of membrane proteins to shared or different subcellular compartments, and the ultimate protein destinations can vary depending on physiological conditions, cell types, developmental expression, and lineages (Gabaldón and Pittis, 2015). Moreover, since membrane proteins are involved in essential cellular pathways, they are often recognized in the pathophysiology of many diseases and are major targets for pharmaceutical agents, with more than 60% of drug targets being membrane proteins (Overington et al., 2006). Hence, investigating the topology, localization, and expression of homologous protein families can provide insight in their different functional activities and identify potential candidates for further studies on drug targets.

The membrane proteome can be categorized based on the number of alpha-helices that span the membrane, with several studies pointing to a correlation between the tertiary structure and functional activities. For example, many receptors are members of the largest human protein family—the 7 TM G protein-coupled receptors (GPCR) that are key drug targets with important roles in mediation of various signals (Lagerström and Schiöth, 2008). Transporters are another well-researched group and tend to have six or more transmembrane helices, such as the solute carriers that contain 10–14 TM regions (Schlessinger et al., 2013) or the major facilitator superfamily that contains 12 TMs (Reddy et al., 2012). Our previous studies have indicated that the group of proteins that contain five transmembrane helices (5TM) include diverse and important families, yet an in-depth analysis of this small class of proteins has not been published.

In this study, we perform comprehensive bioinformatic analyses to identify and characterize the 5TM proteins in the human genome. We collate information on enzyme and transporter classifications, topology, localization, expression, and disease associations to describe the predominant functional activities and possible investigative drug targets with this group of proteins. Further, we examine the major 5TM families and present a new phylogenetic analysis of the sideroflexin family.

## Analysis Results

The 5TM dataset consists of an interesting mix of 58 proteins; ∼60% (35 proteins) are members of 12 families that contain two or more proteins that are predicted to have five transmembrane regions while the other 40% are singlets. Further, 10 of the families (31 proteins) and nine of the singlets appear to be unique protein families with the 5TM architecture without homologues in humans. The inference that the 5TM group contains unrelated protein families comes from literature and database sources as we did not perform phylogenetic analysis on the entire 5TM group (only the sideroflexin family). The other two families and 14 singlets contain Pfam domains or belong to protein families that contain additional members that do not have five membrane regions predicted. Thus the majority of this compact dataset is comprised of complete small families and unique single proteins that contain five membrane-spanning regions, as opposed to say the 7 TM architecture that is primarily comprised of the large homologous GPCR superfamily with 800+ proteins.

The original *Homo sapiens* protein sequences file contained 33,420 total entries including isoforms. The sequences were pre-processed and any predicted signal peptides were removed and the sequences were then evaluated with multiple resources that predict transmembrane alpha-helices. Ambiguous entries, incorrectly predicted proteins, and isoforms were removed while some proteins were manually added through literature research and additional transmembrane prediction resources. The 5TM group tends to be small, with a recent prediction of 93 transmembrane proteins (Dobson et al., 2015), which is comparable to our initial assessment of 106 proteins after removing isoforms of sequences and before manual curation. Our dataset lends toward a conservative estimate of the 5TM group as we sought agreement with multiple transmembrane prediction resources (Fagerberg et al., 2010; Dobson et al., 2015) regarding the accuracy of individual and protein family predictions, particularly if the 3D structure (Berman et al., 2000) has not yet been experimentally determined. While homologous protein families are generally assumed to share similar structures, there is not always prediction agreement on the transmembrane regions in families and we chose the best estimate by comparing different resources. Functional annotations and localization information were compiled through Gene Ontology (GO) descriptions (Binns et al., 2009; The Gene Ontology Consortium., 2019), the Human Protein Atlas (Thul et al., 2017), and the PANTHER classification database (Mi et al., 2019). See Methods for details and Supplementary Figure 1 for an overview of the bioinformatic tools used in the analysis.

### Classification

The dataset is classified into three main functional groups: enzymes, transporters, and proteins that engage in varied functional activities. However, nearly 10% of the proteins are still not fully characterized and their function remains obscure, such as transmembrane protein with metallophosphoesterase domain (TMPPE), solute carrier family 66 member 3 (SLC66A3), and transmembrane protein 41A (TMEM41A).

The enzymes contain 17 proteins with Enzyme Commission (EC) identifiers plus another three proteins are identified with enzymatic activity or members of enzyme families that do not have an associated EC number (Table 1). The enzymes include one protein acting as an oxidoreductase (EC:1); seven acting as transferases (EC:2) including five acyltransferases and two phosphotransferases; seven hydrolases (EC:3) with the majority of them esterases and one dolichyldiphosphatase; and also one lyase (EC:4) and one isomerase (EC:5).

**TABLE 1:** Proteins involved in enzymatic activities.

Protein family (gene name)	EC	Functional activity	Localization
*TLC domain* (TLCD4*, TLCD3A)	2.3.1.-	Ceramide synthesis, possibly involved in lipid trafficking, metabolism, or sensing	ER, Nucleus
AB hydrolase superfamily, Lipase family (DAGLA, DAGLB)	3.1.1.-	Hydrolase activity	PM
Dual specificity phosphatase catalytic domain (TPTE, TPTE2)	3.1.3.-	Phosphatase activity	Golgi apparatus, ER
*Metallophosphoesterase domain* (TMPPE, TMEM62*)	3.1.-.-	Hydrolase activity	Nucleoplasm, Mitochondria
*Emopamil-binding protein family* (EBP, EBPL*)	5.3.3.-	Cholesterol biosynthesis Lipoprotein internalization N.B: EBPL function is undetermined but not involved in cholesterol biosynthesis	ER, PM, Vesicle, Vacuole
CH25H	1.14.-.-	Catalyzes the formation of 25-hydroxycholesterol from cholesterol	ER, Vacuole
ZDHHC4	2.3.1.-	Protein-cysteine S-palmitoyltransferase activity, protein targeting to membrane	Golgi apparatus, ER, Vacuole
AGPAT4	2.3.1.-	Transferase activity, transferring acyl groups	Golgi apparatus, ER, Nucleoli, Vesicles
RNFT1	2.3.2.-	E3 ubiquitin-protein ligase	ER, Nucleoli
SYVN1	2.3.2.-	E3 ubiquitin-protein ligase	ER, PM, Nucleoplasm, Vacuole
CDIPT	2.7.8.-	CDP-diacylglycerol metabolic process	PM, Nuclear membrane
TAOK2	2.7.11.-	Serine/threonine-protein kinase	CM, Nucleoli, Nucleoplasm
AIG1	3.1.-.-	Hydrolase activity: Long-chain fatty acid catabolic process	Golgi apparatus, ER
DOLPP1	3.6.1.-	Hydrolyzes dolichyl pyrophosphate and monophosphate	ER, Vesicle, Vacuole
HACD2	4.2.1.-	Catalyzes reaction in long-chain fatty acids elongation cycle	ER

Asterisk (*) indicates protein is involved in enzymatic activity or member of enzyme family, but does not have an Enzyme Commission (EC) identifier. Families with italic font indicates additional homologous proteins were identified but a different number of transmembrane regions predicted (i.e., more or less than 5TM regions). CM, cell membrane; ER, endoplasmic reticulum; PM, plasma membrane.

The transporters include 16 proteins that have Transporter Classification Database numbers (TCDB) and also an additional nine proteins that function in transport activities but do not have an associated TCDB number (Table 2). The transporter class includes four channels and pores (1.-.-) with three represented by the anion channel tweety family; five electrochemical potential-driven transporters (2.A.-) including three members of the sideroflexin (SFXN) family and the two members of the membrane protein insertase family; one protein is identified as an auxiliary transport protein (8.A.-); and six transporters are involved in incompletely characterized transport systems (9.A/B.-) including three members of the Yip1 domain family (YIPF). One acyltransferase enzyme also acts as a primary active transporter (3.A.-) from the ER retrotranslocon family and one esterase is identified in an incompletely characterized transport system (9B); however, both are labeled as enzymes to prevent redundancy. Additionally, one transporter, TSPO, has also been identified previously as the peripheral benzodiazepine receptor but subsequent studies showed that it is expressed throughout the body and its primary function appears to be involved in cholesterol transport. This was the only protein identified as a receptor. Transporters described with a TCDB identifier may be under-represented as recent work has been published on the sideroflexin (Kory et al., 2018; Rivell et al., 2019) as well as YIPF (Shaik et al., 2019) family members.

**TABLE 2:** Proteins involved in transport activities.

Protein family (gene name)	TCDB	Functional activity	Localization
Tweety family (TTYH1, TTYH2, TTYH3)	1.A.48.-.-	Swelling-dependent volume-regulated anion channel in astrocytes	PM
OXA1/ALB3/YidC family (OXA1L, COX18)	2.A.9.-.-	Insertases: translocation of COX2 and integral membrane proteins	Mitochondria IM
Sideroflexin family* (SFXN1, SFXN2, SFXN3, SFXN4, SFXN5)	2.A.54.-.-	Amino acid transport	Mitochondria IM
TspO/BZRP family* (TSPO, TSPO2)	9.A.24.-.-	Transmembrane signaling Mitochondrial respiration Cholesterol transport	ER, Vesicle, Vacuole Mitochondria OM
YIF1/YIP1 family* (YIF1A, YIF1B, YIPF1-7)	9.B.135.-.-	COPII-coated ER to Golgi transport vesicle-mediated transport	Golgi apparatus, ER
CD47	1.N.1.-.-	Cell adhesion, membrane transport	PM, Vesicle
STIMATE	8.A.65.-.-	Calcium channel regulator activity	ER, Vacuole
ARV1	9.A.19.-.-	Cholesterol transport	ER, Vesicle,
TMEM41A	9.B.27.-.-	Putative transport protein; metastasis via modulation of E-cadherin	ER, Golgi apparatus

Asterisk (*) indicates a family member is presumably involved in transport activity although it does not have an TCDB identifier associated with it. ER, endoplasmic reticulum; IM, inner membrane; OM, outer membrane; PM, plasma membrane; TCDB, Transporter Classification Database.

The third major class is engaged in varied functional activities which includes 25 proteins (Table 3). This class includes two complete families that contain the 5TM architecture: Dual oxidase maturation factor 1 and 2 (DUOXA) and also the Prominin-1 and –2 (PROM) families. Four single proteins with 5TM regions were identified that do not have any homologous family members in humans: TEX261, TMEM79, UNC50, and the fusion protein CHRNA7-FAM7A. And five more single proteins were identified that are members of families that contain proteins with varying number of TM regions predicted.

**TABLE 3:** Proteins with the 5TM architecture that perform varied functional activities.

Protein family (gene names)	Functional activity	Localization
Dual oxidase maturation factor family (DUOXA1, DUOXA2)	Transport of DUOX1/2 from ER to PM	ER, PM
Prominin family (PROM1, PROM2)	Cholesterol binding	PM, Vesicle, Nucleoplasm
ATP6V0B	Proton-conducting pore forming subunit of vacuolar ATPase	Vacuole
BFAR	Apoptosis regulator	ER, PM
CLPTM1	May play a role in T-cell development	ER, PM
CHRFAM7A	Transmembrane signaling receptor activity, regulation of membrane potential	PM
DNAH3	Cilium-dependent cell motility	Plastid
SLC66A3	Possible transport of amino acids across the lysosomal membrane	ER
TEX261	COPII-coated ER to Golgi transport vesicle	Vesicle, Vacuole Nucleoplasm
TMEM79	Regulated exocytosis, cornification	Golgi apparatus
UNC50	Protein transport	Vacuole, Golgi apparatus

*ER, endoplasmic reticulum; PM, plasma membrane.*

### Topology

**FIGURE 1:**
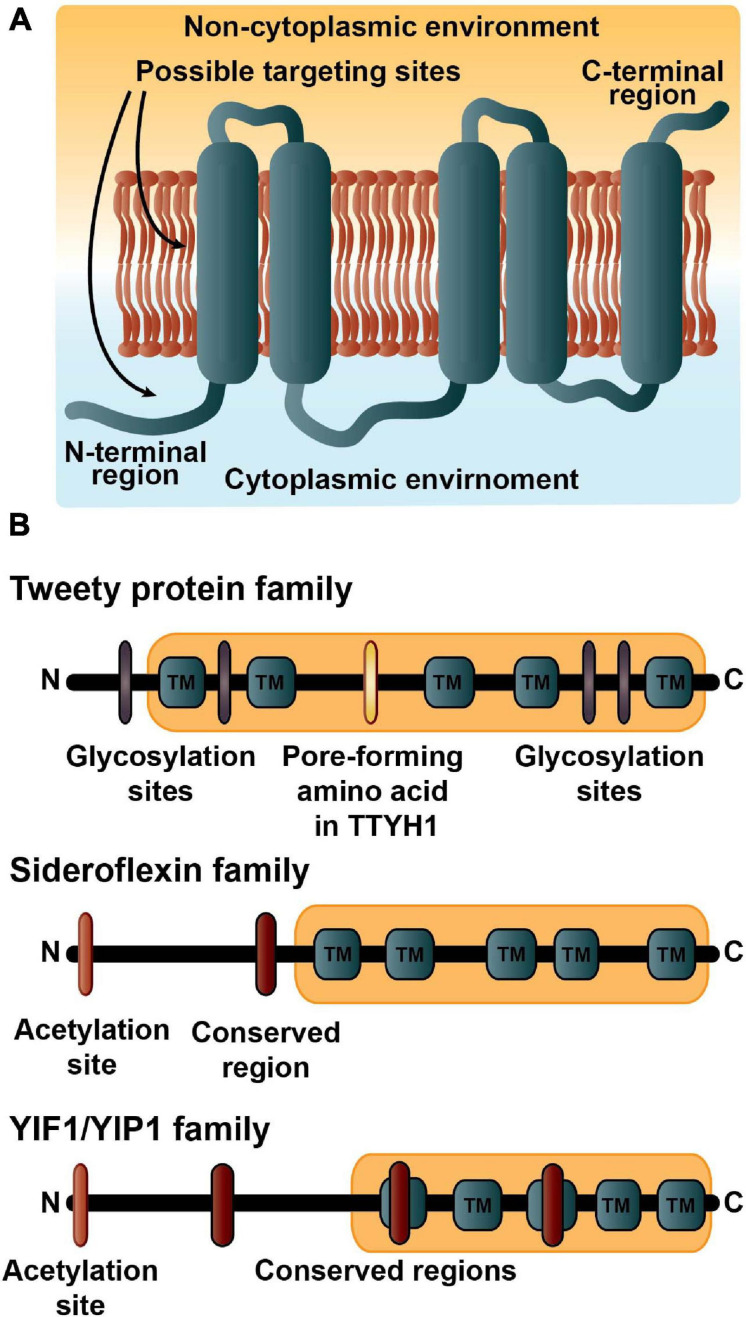
The five transmembrane architecture. **(A)** The basic topology of the 5TM dataset. More than half the proteins in the dataset have the amino (N)-terminal region in the cytoplasmic environment and the carboxyl (C)-terminal in the luminal region. Many of the proteins are expected to contain targeting signals embedded in the first transmembrane region along with possibly amino acid residues in the N-terminus. **(B)** The domain structures and important residue modifications affecting localizations of the three major 5TM families. The description of the tweety family includes estimates of four possible glycosylation sites in purple; the important pore-forming amino acid (R165) in TTYH1 indicated in yellow (Han et al., 2019); and the Pfam tweety domain (PF04906) in light orange. The Sideroflexin family is annotated with a possible acetylation site at residue one or two and colored orange; the conserved HPDT residues are the red symbol; and the sideroflexin Pfam domain (PF03820) is in light orange. Many of the YIPF proteins have an acetylation site at residue one or two that is colored orange; three conserved motifs are indicated in red; and the YIPF Pfam domains (PF03878 and PF04893) are shown in light orange.

We analyzed if there were any associations between functional activities and membrane topology, meaning if the amino (N)-terminal laid in the cytoplasmic environment (also known as in) and hence the carboxylic (C)- terminal in the lumen or non-cytoplasmic region, or if the N-terminal was found in the non-cytoplasmic region (out) with the C-terminal in the cytoplasmic environment (Figure 1). The results from TOPCONS2 and experimental evidence yielded 30 proteins with the N-terminal in the cytoplasmic region and 28 proteins with the N-terminal in the lumen or non-cytoplasmic region. Of the 17 enzymes, only the six carboxylic ester hydrolases have an N-terminal within the cytoplasmic environment with the C-terminal inside the lumen. The 16 transporters have 11 N-terminals in the cytoplasmic environment and five N-terminals in the lumen region. The tweety, sideroflexin, and YIPF families, whether identified with a TCDB number or not, have their N-terminals in the cytoplasmic region while the membrane protein insertase family have N-terminals in the non-cytoplasmic environment. The 25 proteins with varied functions have a relatively even split with 13 N-terminals in (including eight from the aforementioned sideroflexin and YIPF members) and 12 N-terminals out.

### Localization

Only 12% (7 proteins) in the dataset are predicted to contain an amino-terminal signal sequence using SignalP v5.0 (Armenteros et al., 2019b), which is not unusual as many membrane proteins use the first hydrophobic transmembrane sequence to direct protein translocation (Rapoport, 2007). In contrast to soluble proteins that use a signal sequence and subsequently have their N-terminus in the cytosol, the N-terminus of a transmembrane proteins can reside on either side of the membrane depending on the amino acid composition of the first transmembrane segment (Rapoport, 2007). Furthermore, protein modifications such as glycosylation and acetylation can also affect subcellular targeting (Ree et al., 2018) and topogenesis (Goder et al., 1999), although they may not be the sole determining factor for protein targeting (He et al., 2008). N-GlyDE (Pitti et al., 2019), an N-linked glycosylation site prediction resource, was used to assess possible glycosylation sites in the 5TM dataset. Nearly one-third (19 proteins) were predicted to contain N-linked glycosylation sites, and 12 of the proteins had multiple sites predicted. Thirteen proteins were identified with having N-terminal acetylation sites at position one or two from UniProt (UniProt Consortium, 2019). TargetP v2.0 (Armenteros et al., 2019a) was used to assess if N-terminal pre-sequences such as mitochondrial transit peptides existed in our proteins, although except for one borderline case, there were not any mitochondrial targeting peptide sequences found in the dataset. Instead it appears that the targeting signal might be embedded in various regions of the protein, for example in the first transmembrane domain along with amino acid residues in the N-terminus, as found in SFXN2 (Mon et al., 2019).

**FIGURE 2:**
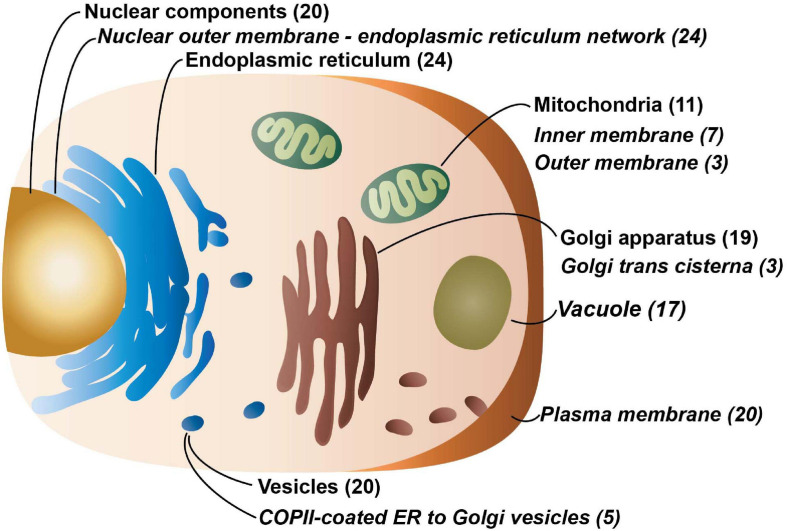
The major cellular localizations of the 5TM proteins. Localization information and analysis for with the number of proteins identified for each locale is in parenthesis and compartments that are overrepresented in comparison to the human transmembrane proteome are indicated in italics. Proteins that localize to the nuclear outer membrane-endoplasmic reticulum network, the inner and outer membrane of the mitochondria, the Golgi trans cisterna, vacuoles, the plasma membrane, and COPII-coated ER to Golgi vesicles are over-represented. Data for this figure is solely from the PANTHER Classification System and the overrepresentation analysis is from the PANTHER Overrepresentation Test (v14.1) (Mi et al., 2019) with the Gene Ontology (GO) Annotation database released on 2019-07-03. Fisher’s Exact test was performed and the False Discovery Rate was calculated with *p* < 0.05. The human transmembrane protein identities are from Attwood et al. (2017). 5,725 out of 5,779 proteins were successfully mapped while 55 of 58 proteins from the 5TM dataset were successfully mapped using GO annotation.

The Cell Atlas (Thul et al., 2017), which uses antibody-based profiling by immunofluorescence confocal microscopy and currently covers 12,390 genes, as well as the PANTHER Classification System (Mi et al., 2019) was used to assess the localization of the 58 proteins in the dataset (Figure 2). The most common area the proteins were localized to was the nucleus and associated structures (20 proteins) including the nuclear membrane, nucleoli, and nucleoplasm. There is evidence that nine proteins localize to the Golgi apparatus; additionally, Gene Ontology (GO) annotation, which is based on different types of evidences (see “Materials and Methods” for details), describes 10 additional proteins that localize there. There is also evidence from the Cell Atlas that nine proteins are found in vesicles with an additional 11 GO annotated that localize to vesicles. Eight proteins have evidence from Cell Atlas they localize to mitochondria with an additional three annotated to be found there. While only six proteins have evidence of localizing to the endoplasmic reticulum (ER), an additional 12 are GO annotated to be found there and a further fourteen are predicted to localize there according to DeepLoc—v1.0 (Almagro Armenteros et al., 2017). And six proteins also have evidence from Cell Atlas that they localize to the plasma membrane while GO annotation notes another fourteen.

## The Three Major 5TM Families

Nearly one-third of the 5TM dataset consists of three main families: the sideroflexin, tweety and YIP1 domain family (YIPF) proteins. Hence, with such a heavy influence on possible functional activities and localization preferences associated with the 5TM architecture, we performed more comprehensive analysis of these families. Additionally, we performed phylogenetic analysis on the sideroflexin family as we were not able to find a current wide ranging evolutionary study on this family while investigations have been published on the tweety and YIPF families (Matthews et al., 2007; Han et al., 2019; Shaik et al., 2019).

### The Sideroflexin Family

The sideroflexin (SFXN) protein family contains five homologues in humans: SFXN1, SFXN2, SFXN3, SFXN4, and SFXN5. Interest in this family is burgeoning as SFXN1 and SFXN3 (and perhaps SFXN2) have been recently identified as the main mitochondrial serine transporters required for one-carbon metabolism needed for biosynthesis (Kory et al., 2018). Sideroflexin members contain the mitochondrial tricarboxylate/iron carrier conserved domain (PF03820/IPR004686). All members of this family share the same N- and C-terminal topology with the N-terminal inside the cytoplasmic region and the C-terminal outside the cytoplasmic region in the lumen. Additionally, SFXN1-4 have post-translational acetylation at either N-terminal position one or two (UniProt Consortium, 2019), which can affect translocation as well as protein stability and degradation (Arnesen, 2011). From studies in *Xenopus* embryos, sideroflexin homologs are found throughout the body and have both overlapping and non-overlapping expression in different tissues (Li et al., 2010). Furthermore, as the sequences and structures of the sideroflexin homologues are similar, there are some functional redundancies among them. Notably, each of the sideroflexin homologues has been shown to be involved in different disease pathologies, making them possible therapeutic targets.

**FIGURE 3:**
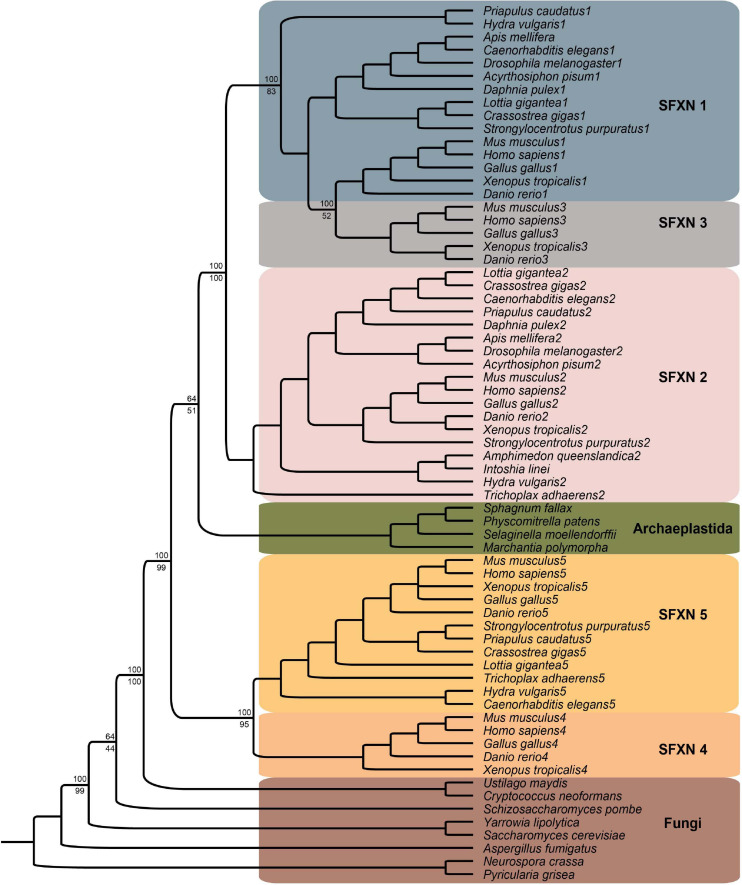
Phylogenetic analysis of Sideroflexin family. Phylogenetic reconstruction is the result of Bayesian inference posterior probabilities and bootstrapping analysis with the best-scoring maximum likelihood tree using RAxML (v8.2.10) (Minjarez et al., 2016) on 30 taxa with 67 sequences. Support values are given in percent at the nodes of the major clades differentiating the sideroflexin gene families. The protein sequences were aligned using Mafft (v6) (Fang et al., 2018) with E-INSI-I algorithm and JTT substitution model. MrBayes was used with amino acid mixed model run for 1,000,000 generations. The PROTGAMMAAUTO model in RAxML was used with 500 bootstrap replicates.

A representative phylogenetic tree of the sideroflexin family is shown in Figure 3 and includes 67 sequences from 30 taxa throughout Eukaryota including Metazoa (18 species); Holomycota (8) including Ascomycota, Basidiomycota, Mucoromycota, and Chytridiomycota fungi; and Archaeplastida (4 species). Homologues of SFXN1–5 were identified using BLASTp searches in NCBI databases and then sequences were downloaded. The BLASTp results show that sideroflexin homologues have ancient evolutionary origins in eukaryotes and are found in heterokonta, excavata, archaeplastida, uniconta including amebozoa and opisthokonta, as well as metazoan lineages (not all species shown on figure). While there was one significant hit identified in an archaeon lineage (phyllosphere metagenome), exhaustive searches in other archaeon lineages did not yield any other hits and also this genome was not yet assembled, so it is possible that this hit is an artifact, perhaps due to contamination. Our results corroborate previous studies (Miotto et al., 2007; Li et al., 2010) that SFXN1 and SFXN3 are more closely related to each other than to the other sideroflexins, with SFXN2 being the next most closely related. The sequences from archaeplastida appear to resolve between the SFXN1/SFXN3/SFXN2 and SFXN5/SFXN4 clades, although support values are low for this placement. The fungi lineages form more distant groups basal to the other groups.

#### SFXN1

SFXN1 localizes to the inner mitochondrial membrane (Kory et al., 2018) and is expressed ubiquitously throughout tissues, with the highest amount found in the liver, blood, and kidneys (Fleming et al., 2001; Uhlén et al., 2015). SFXN1 is one of the primary transporters of serine into mitochondria, where it is converted into glycine and formate that are needed for use in one-carbon metabolism (Kory et al., 2018). In dividing mammalian cells, the mitochondrial metabolism of serine is the primary supplier of the one-carbon units needed for biosynthesis of, for example, nucleotides and lipids (Kory et al., 2018). SFXN1 may also transport other amino acids including alanine, glycine, and in particular cysteine. One-carbon metabolism also generates purine synthesis which contributes to proliferation of cancer cells, and SFXN1 has been found to be expressed in many cancers. SFXN1 as well as SFXN2 and SFXN3 are regulated by the Myc transcription factor and may be involved in cancer cell growth in not yet investigated ways. Additionally, SFXN1 has been found to be sub-expressed in brains with Alzheimer’s disease; however, the role of this protein in this neurodegenerative disease remains unknown (Minjarez et al., 2016). Studies in surgical menopause rat models showed changes in brain regions related to depression and dementia also had decreased levels of SFXN1 (Fang et al., 2018).

#### SFXN2

Unlike SFXN1 and SFXN3, SFXN2 localizes to the outer mitochondrial membrane (Mon et al., 2019). It is expressed in the liver and kidney and lowly expressed in other tissues (Fleming et al., 2001; Uhlén et al., 2015) and has been found to be expressed in the developing pancreas in studies of the developmental expression of sideroflexin family genes in *Xenopus* (Li et al., 2010). It is involved in mitochondrial iron homeostasis by regulating heme biosynthesis, however, it is not involved in iron-sulfur cluster assembly like SFXN4 (Mon et al., 2019). Additionally, it may function as a mitochondrial serine transporter, or perhaps provide redundancy for SFXN1 or SFXN3 in this capacity (Kory et al., 2018). As mentioned previously, it may also play not yet investigated roles in cancer cell growth.

#### SFXN3

SFXN3 is localized exclusively in the mitochondria, and in particular the inner mitochondrial membrane (Amorim et al., 2017). It is highly enriched in the brain and is also present in liver, kidney and placenta (Uhlén et al., 2015; Amorim et al., 2017). It is developmentally expressed in neurons, with initial low protein levels in the cortex and hippocampus at birth but then increases as neurons mature and sustains high levels in mature brains (Rivell et al., 2019). SFXN3 is a downstream target of α-synuclein in synapses and is involved in the regulation of synaptic morphology (Amorim et al., 2017). Additionally, it is also one of the main mitochondrial serine transporters involved in one-carbon metabolism (Kory et al., 2018). A study on the roles of the sideroflexin family in pancreatic islets resulted in upregulation of SFXN3, SFXN2, and SFXN5 in diabetic islets, which suggests their function may be related to the regeneration of pancreatic endocrine cells (Yoshikumi et al., 2005). SFXN3 may be implicated in several pathologies. Serine transport may support cancer cell proliferation through the synthesis of nucleotides and one-carbon metabolism (Labuschagne et al., 2014), hence SFXN3 may be critical for cancer growth. And in fact, a patent application was filed in 2017 for a therapeutic antibody that targets SFXN3, which has been shown to be present in tumor-associated macrophages, and strongly reduces the leukemic B cells number (Poupot et al., 2020).

#### SFXN4

As with all sideroflexins, this protein localizes to the mitochondria, with evidence it is expressed specifically in the mitochondrial inner membrane (Hildick-Smith et al., 2013; Paul et al., 2019). It has high protein expression in muscles (Uhlén et al., 2015) and the highest mRNA expression particularly in kidney, brain and heart tissues (Fleming et al., 2001). SFXN4 has unique functional activities including iron-sulfur cluster biogenesis that are components of electron transfer proteins, cellular iron homeostasis, and mitochondrial respiration (Paul et al., 2019). It is implicated in mitochondrial disorders, and mutations in it cause severe complex I deficiency, macrocytic anemia, and optic nerve hypoplasia (Sofou et al., 2019).

#### SFXN5

This protein is also detected in mitochondria, presumably the inner mitochondrial membrane (Miyake et al., 2002), and also the nucleoplasm (Uhlén et al., 2015) and expresses low levels in fetal brain, liver, and kidney tissues with ubiquitous expression at higher levels in all regions of the adult brain (Lockhart et al., 2002). SFXN shows citrate transport activity in rats, where it is specifically expressed in the brain and localizes to the inner mitochondrial membrane of Bergmann glial cells (Miyake et al., 2002). And due to the specialized expression in the brain, it is possible that SFXN5 has undergone neo-functionalization and performs a specific, yet undetermined, task in the cerebral cortex.

### The Tweety Family

The Tweety-homologue Family contains three members: TTYH1, TTHY2, and TTHY3 that have also been the focus of recent investigations. These transmembrane proteins were recently found to be the pore-forming subunits of the swelling-dependent volume-regulated anion channel (VRAC_s__well_) in astrocytes (Han et al., 2019). Volume regulation in the brain is critical for the proper function and health of the nervous system and is regulated by astrocytes as they have high and exclusive expression of the aquaporin-4 (AQP4) water channel (Han et al., 2019). VRAC_s__well_ is activated by AQP4-dependent swelling and hence the tweety homologues are crucial components in maintaining nervous system health. Consequently, VRAC_s__well_ has been associated with several pathophysiological conditions such as cerebral edema following excessive oxidative stress, ischemia, traumatic brain injury, and glioma (Han et al., 2019). As might be expected due to their functional activities in astrocytes, both TTYH1 And TTYH2 have tissue enriched mRNA expression in the cerebral cortex (Uhlén et al., 2015). Furthermore, expression of TTYH2 is upregulated in renal cell carcinoma (Rae et al., 2001) and also significantly upregulated in colon cancer cell lines (Toiyama et al., 2007).

The precise topology of each of the tweety homologues has varied including 6 TM with the N- and C-terminals on the same side of the membrane (Suzuki and Mizuno, 2004); 5TM with the N-terminus extracellularly and C-terminus within the cytoplasm (Campbell et al., 2000; Rae et al., 2001); and most recently 4 TM (although possibly 5TM; TTYH1) and 5TM (TTYH2 and TTYH3) with the N-terminal in the cytoplasmic region and the C-terminal also cytoplasmic (TTYH1) or extracellularly (the other two) (Han et al., 2019). All three tweety homologues in our dataset had 5TMs predicted with the N-terminals in the extracellular environment, although due to this latest evidence we categorized them as laying in the cytosolic region. Four glycosylation sites are also described on several tweety homologues (Figure 1B).

### The YIP1 Domain Family (YIPF) Proteins

Nine human members make up this family including YIF1A, YIF1B, YIPF1, YIPF2, YIPF3, YIPF4, YIPF5, YIPF6, and YIPF7. These proteins form complexes with each other in specific partner pairs to form oligomers with 20 transmembrane segments (Shaik et al., 2019). All of the proteins except YIPF7 and YIF1A appear to have low tissue specificity and are detected in all the investigated tissues, while YIPF7 is enriched in skeletal muscles and tongue and YIF1A is tissue enhanced in the liver and both are detected in some other tissues. All of the YIPF members are expected to localize to the early, middle or late compartments of the Golgi apparatus, depending on the protein complexes that are formed, and transport to and from between other compartments (Shaik et al., 2019). Hence, additional annotations describe YIF1B, YIPF4, YIPF5, and YIPF6 to be found in vesicles, while YIPF1, YIPF3, and YIPF5 have also been found in the nucleoplasm. A recent review summarized that the proteins appear to have overlapping functions, including ER to Golgi transport as well as intra-Golgi transport at the vesicle docking/fusion stage (Yang et al., 1998; Matern et al., 2000) and roles in the membrane trafficking pathway, although the exact functions of several of the specific complexes are still in debate (Shaik et al., 2019). YIPF proteins are implicated in various disease pathways as well: YIPF4 interacts with several different types of human papillomaviruses (HPV), however, its relationship with HPV is inconclusive and further studies are needed to examine the change in YIPF4 expression during keratinocyte differentiation and the presence of viral proteins (Shaik et al., 2019). Increased expression of YIPF6 in prostate cancer cells that showed bone metastasis and castration resistance has also been reported (Djusberg et al., 2017), although if or how this may contribute to the malignant phenotype of the cancer cells is not clear. YIF1A and possibly YIF1B interact with VAPB and its mutant VAPB-P56S, which has been linked to motor neuron degeneration in amyotrophic lateral scleroses type 8, indicating the interactions of the YIPF proteins with VAPB may have a significant role in the pathology of the mutant VAPB (Kuijpers et al., 2013).

### Proteins Associated With Diseases

In addition to the tweety, sideroflexin, and YIPF families’ associations with diseases just discussed, six proteins in the dataset had strong associations with diseases using the DisGeNET resource (Piñero et al., 2017; see Supplementary Table 1). Both ARV1 and EBP are associated with intellectual disabilities as well as epilepsy in the former and cataracts in the latter. DAGLA was associated with neurodegenerative disorders while DUOXA2 is associated with thyroid issues, which is discussed below. The sideroflexin family and OXA1L from the mitochondrial inner membrane protein family, which localize to the mitochondria, also have proteins associated with mitochondrial diseases.

## Discussion

### Primary Functional Activities

While the different groups of the 5TM do not share common descent, there are two overarching functional themes. These are the *establishment of localization*, where many of these proteins are involved in different processes that localize a substance or cellular component through movement, tethering, or selective degradation. The other main function is *transporter activity* in which proteins are described as enabling the directed movement of substances into, out of or within a cell or between cells (The Gene Ontology Consortium., 2019). More than half of the dataset (30 proteins) are annotated with establishing localization of other substances or proteins, although this may be an underestimate of the number of proteins involved as some members of the YIPF family, for example, are predicted to be involved in ER-to-Golgi as well as intra-Golgi transport at the vesicle docking/fusion step and are not described with this GO term. The two members of the DUOXA family are an interesting example of localization activity; DUOXA2 (and presumably DUOXA1) are ER-resident proteins that allow ER-to-Golgi transition, maturation, and translocation to the plasma membrane of functional DUOXA2 (and DUOXA1), which are essential components in generating thyroid hydrogen peroxide for hormone synthesis at the apical membrane (Grasberger and Refetoff, 2006). And DUOXA2 is also associated with thyroid disorders, including congenital hypothyroidism and thyroid agenesis (Piñero et al., 2017). Six of the singlet proteins without apparent paralogues in humans are also described with localization activities: ARV1, CD47, CHRFAM7A, TEX261, TMEM79, and UNC50 function in sterol distribution from ER to the plasma membrane, cell migration and adhesion, exocytosis, and vesicle transport among other activities.

An interesting aspect of the 5TM group is that it is polyphyletic, i.e., composed of multiple small protein families as well as singlet protein families (without human homologues) that do not appear to share a recent common single ancestor, yet many of these protein families perform similar functional activities. How exactly the 5TM architecture contributes to the ubiquitous functional activities of this group was not able to fully elucidated, as factors such as the topology of the N- and C- terminals, which contribute to activities, were evenly divided between the cytoplasm and non-cytoplasmic environments. Within the human transmembrane proteome, transmembrane groups categorized by their number of transmembrane regions can be comprised of differing numbers of evolutionarily related (or unrelated) protein families. For example, the 1 and 2 TM groups are two of the largest classes and encompass many unrelated protein families that range in size from 1 to ∼150 proteins that often participate in varied functional activities (Almén et al., 2009; Sällman Almén et al., 2012). This is in contrast to, for example, the 10 and 12 TM groups that contain the large (∼400 proteins) solute carrier (SLC) transporter family (Almén et al., 2009; Perland and Fredriksson, 2017) or the well-known 7 TM receptor group that contain the ∼800 proteins of the GPCR family (Lagerström and Schiöth, 2008). Hence the 5TM group, while being composed of multiple unrelated protein families that do not share the same topology, is unique in that the described localization and transporter activities are prevalent for this group. When we collate GO annotations, experimental evidence, and TCDB descriptions, there are approximately 20 5TM proteins that are characterized as being involved in transport activities and nearly half of them localized to mitochondria. Proteins involved in mitochondrial transport (8 proteins) and in particular proteins that are integral components of the mitochondrial inner membrane are overrepresented in comparison to the entire *Homo sapiens* transmembrane proteome (Fold Enrichment (FE) = 9.72; FDR = 2.71e-02 and FE = 12.23; FDR = 3.70e-04, respectively). This includes the five members of the sideroflexin family, the two members of the mitochondrial inner membrane protein family (COX18 and OXA1L) as well as TSPO. The important sideroflexin family that acts in amino acid transport into the mitochondria was described previously. OXA1L and COX18 (OXA1L2) act as membrane insertases: OXA1L functions in the biogenesis of membrane proteins and the insertion of integral membrane proteins into the mitochondrial inner membrane (Haque et al., 2010) and COX18 facilitates the translocation of COX2 across the mitochondrial inner membrane (Bourens and Barrientos, 2017). TSPO is engaged in mitochondrial cholesterol trafficking (Taylor et al., 2014). And this category may be underestimated as well, for example TSPO2, homologue to TSPO and not annotated as involved in transporter activity, is suggested to have become sub-functionalized and is involved with cholesterol trafficking and redistribution during erythropoiesis with ER and nuclear membrane localization (Fan et al., 2009).

An interesting aspect to the 5TM dataset is that only the three members of the tweety family are annotated to form homo and/or heteromeric subunits to create an actual pore for solutes to cross membranes (Han et al., 2019), while virtually all other transport activity involves membrane trafficking, vesicle-mediated transport, or protein translocation across membranes. However, while three members of the YIPF family are identified in the TCDB with vesicle-mediated transport activities, all nine members are hypothesized to function as channels, transporters, or possibly even transmembrane receptors as at least four YIPF molecules associate to form higher order oligomers with 20 transmembrane regions, which is highly suggestive of transport activity (Shaik et al., 2019). With the exception of these two families, the 5TM architecture appears to facilitate transport mechanisms, for example vesicle budding and trafficking or insertase activities that allow movement across membranes, rather than forming oligomeric complexes that create a pore or channel for the transport of substances.

### Localization Destinations Associated With Functions

Nearly 30% of the 5TMs (16 proteins) localize to vesicles, including five proteins that function in COPII-coated ER to Golgi transport vesicles, which is also over-represented in our 5TM dataset in comparison to the human membrane proteome (FE = 9.22; FDR = 1.70e-02). This includes four members of YIPF including YIF1A, YIF1B, YIPF5, YIPF6, and TEX261, which is a unique 5TM singlet family without any other human homologues identified. And in fact, TEX261 plus YIF1A, YIF1B, and UNC50, also a 5TM singlet family, are integral components of the Golgi membrane, which is also over-represented (FE = 13.28; FDR = 2.90e-02).

More than 40%—24 proteins—localize to the nuclear outer membrane-endoplasmic reticulum membrane network and are also over-represented in comparison to the human transmembrane proteome (FE = 2.84; FDR = 1.80e-04). This might be expected due to the predominant activities such as protein transport and membrane trafficking. In fact, more than half of these proteins are also found to localize to other sub-compartments as well, including the Golgi apparatus, vesicles, and vacuoles. The varied 17 proteins that localize to vacuoles are over-represented as well (Fe = 2.93; FDR = 7.12e-03). Vacuoles have a variety of different functions such as storage, structural support, exocytosis, growth, and isolation of various substances. Included in the dataset is ATP6V0B, which is a subunit in an enzyme complex that mediates acidification of intracellular compartments, including vacuoles.

### Phylogenetic Analysis

We analyzed the evolutionary origins of the three major families. A recent comprehensive analysis of YIPF proteins concluded that homologues of YIPF proteins have deep evolutionary origins and can be found in bacteria, archaea, and also throughout eukaryotic species including excavate, SAR, archaeplastida, uniconta and metazoan lineages (Shaik et al., 2019). In comparison, the tweety family shows evolutionary origins within eukaryotes with homologues found in amoebozoa, archaeplastida, and in different metazoan lineages (Matthews et al., 2007). Our phylogenetic analysis of the sideroflexin family also shows evolutionary roots within eukaryotes, while there were not any homologues identified in bacteria or archaea. Hence, it appears that the YIPF proteins are the most ancient of the three major families identified in the 5TM dataset. Further, we did not find any evidence of common decent of these families even though they all contain the rather rare 5TM architecture.

### Important Roles as Cancer Prognostic Markers

**FIGURE 4:**
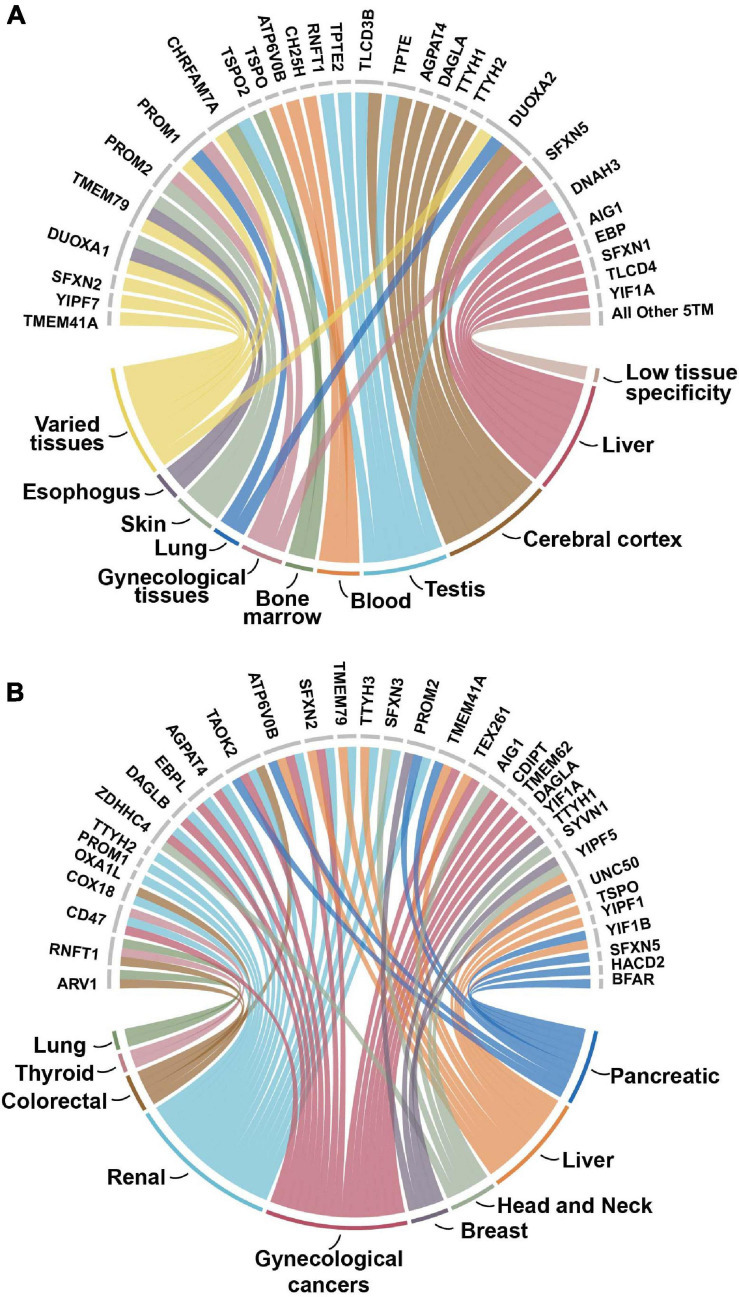
**(A)** Enhanced tissue expression of 5TM dataset. The enhanced or enriched expression of proteins in the 5TM dataset with the different types of tissues on the bottom part of the figure and associated proteins on the top part. Data are from The Tissue Atlas (Amorim et al., 2017). More than 35% of the proteins have enhanced or enriched expression in the cerebral cortex, liver, testis and blood tissues. The category Varied tissues includes intestine, breast, thyroid, parathyroid, gall bladder, prostate, and pancreas tissues. The category All Other 5TM includes thirty proteins in the dataset that have low tissue specificity. **(B)** 5TM proteins as prognostic markers for cancer. The nine different tumor types are on the bottom part of the figure while the 35 prognostic proteins associated with them are on the top half. Approximately 60% of the genes in the dataset are identified in the Pathology Atlas as candidate prognostic genes that are associated with the clinical outcome of different tumor types. The genes are identified from correlation analyses of gene expression and clinical outcome where Kaplan-Meier plots with high significance (*p* < 0.001) were considered prognostic (Pujar et al., 2018). Of the 35 proteins identified, 21 are associated with several different types of cancers. Gynecologic cancer includes cervical, endometrial, ovarian, and urothelial cancers. Proteins not identified as prognostic are not included in the figure.

While less than 10 proteins are identified in gene-disease associations, roughly 60% are identified as cancer prognostic markers according to The Pathology Atlas, where candidate prognostic genes are associated with clinical outcome of different tumor types (Uhlen et al., 2017). Of these 35 proteins, 21 of them are prognostic for several different types of cancers and 14 were associated with just one tumor type (Figure 4). Renal, gynecological, and liver cancers were the most common types of cancers associated with this dataset. While there were not any overarching patterns in protein families with cancer prognosis, two of the three tweety family as well as two of the five sideroflexin members were prognostic in renal cancer.

## Conclusion

This analysis characterizes the 58 proteins, including the 10 unique families and nine singlet proteins, that contain the five transmembrane alpha-helical architecture from the human proteome. Proteins that localize to the nuclear outer membrane-endoplasmic reticulum network, the inner and outer membrane of the mitochondria, the Golgi trans cisterna, vacuoles, the plasma membrane, and COPII-coated ER to Golgi vesicles are over-represented in comparison to the human transmembrane proteome. Furthermore, this group of proteins is predominantly involved in localization activities through movement or tethering of cell components and transport mechanisms including protein targeting and transport, and membrane trafficking. The three larger families including the tweety, sideroflexin, and YIPF families, which comprise almost 30% of the dataset, are heavily engaged in these activities. Furthermore, interest in these three families is currently piqued due to recent discoveries into their important functional activities. Nearly 30% of the proteins in the 5TM dataset show enhanced expression in the cerebral cortex, liver, or testis. Notably, ∼60% of the proteins are identified as cancer prognostic genes that are associated with different clinical outcomes of different cancer types, indicating the value in continued investigation in 5TM families. As up to 10% of the 5TMs are still not fully described, much work is still needed to clarify the functional properties of several of the 5TM members, for example TMPPE, SLC66A3, and TMEM41A. Overall, this work provides an overview of the functional properties of the 5TM proteins and adds to the understanding of the global diversity among transmembrane proteins.

## Materials and Methods

### *Homo Sapiens* Proteome

The *Homo sapiens* protein sequences with Consensus Coding Sequence (CCDS) annotations of the GRCh38p12 assembly were downloaded from the National Center for Biotechnology Information (NCBI) (Pujar et al., 2018). The CCDS annotations were used as coordinated manual reviews and updates by expert curators are used to annotate the gene set. The Havana group at EMBL-EBI and the RefSeq team at NCBI reconcile the main manual curation while the automatic methods are coordinated by the Ensembl group and NCBI genome annotation computational pipeline. The dataset originally contained 33,420 entries which included alternatively spliced sequences from the same gene.

### Transmembrane Topology Prediction

Similar to alpha-helical transmembrane helices, cleavable signal peptides (SP) share common hydrophobic compositions that can make discernment between the two elements difficult for transmembrane prediction algorithms. Hence, the software SignalP v5.0 was used to predict the presence of signal peptides in the dataset. The default parameters were used with the organism group as *eukarya*. The mature sequences with the signal peptides removed were collated with the rest of the dataset. TOPCONS-single webserver was used to predict membrane protein topology as it is appropriate to use for full proteome scans (Hennerdal and Elofsson, 2011). To achieve greater accuracy, a consensus or majority decision method using several different algorithms is recommended. This software incorporates four default multiple transmembrane prediction methods into a hidden-Markov model that estimates the consensus topology for a predicted transmembrane protein (Hennerdal and Elofsson, 2011). The default software includes: SCAMPI-single (Bernsel et al., 2008); S-TMHMM (Viklund and Elofsson, 2004); HMMTOP (Tusnády and Simon, 2001); and MEMSAT (Jones et al., 1994). The resulting proteins identified as 4, 5, and 6 TM were retrieved, which included 1,529 sequences that constituted both canonical sequences as well as alternative spliced gene products. These sequences were then assessed with TOPCONS2, which is another consensus transmembrane prediction software that contains a different set of methods: OCTOPUS (Bernsel et al., 2008); Philius (Reynolds et al., 2008); PolyPhobius (Käll et al., 2005); SCAMPI (Bernsel et al., 2008); SPOCTOPUS (Viklund et al., 2008), and ΔG-scale (Hessa et al., 2007). TOPCONS2 incorporates scans of homology-based databases such as Pfam (El-Gebali et al., 2019) and the Conserved Domain Database (CDD) (Marchler-Bauer et al., 2017) to help determine hits more accurately and faster. As the performance for the best prediction methods for eukaryotic membrane proteins has been estimated to be between 60 and 70% (Tsirigos et al., 2012), the 211 sequences that were predicted to contain five transmembrane regions were then further manually curated. The sequences were assessed as either canonical or alternatively spliced using UniProt, where the canonical sequence is determined as the most prevalent, the most similar to orthologous sequences, the composition of the amino acids in the sequence, or typically the longest sequence (UniProt Consortium, 2019). The canonical sequences were chosen to remain in the dataset for further evaluation. As protein families are generally assumed to share similar structures, if one or more members of a family were identified to contain 5TM then other members of that family were also evaluated. To achieve as accurate a dataset as possible, we sought prediction agreement using literature searches that describe experiments that help determine membrane topology, databases that contain experimentally determined structures (Berman et al., 2000), and multiple transmembrane prediction resources (Fagerberg et al., 2010; Dobson et al., 2015).

We compared the sequences in our dataset to the Human Transmembrane Proteome (HTP) database and also evaluated their 92 proteins predicted to be 5TM (Dobson et al., 2015). HTP uses consensus methods with homology scans of different experimentally determined and predicted structures to increase the accuracy of their results. We also compared our sequences to the majority-decision based transmembrane predictions in the Human Protein Atlas^1^ (Fagerberg et al., 2010). Literature searches also aided in confirming (or not) and identifying possibly missed proteins with 5TM topology. The final dataset consists of 58 proteins.

### Annotation, Localization, and Enrichment Analyses

Universal Protein resource, UniProt, was used to provide protein annotations for the dataset and included: review status, Transporter Classification number, Enzyme Commission number, Gene Ontology annotation terms, subunit interactions, post-translational residue modification information, and protein family information (UniProt Consortium, 2019). The DisGeNET drug encyclopedia was also utilized to assess gene-disease associations and the evidence metric *strong* were used to identify relevant associations (Piñero et al., 2017).

The Cell Atlas (Thul et al., 2017) uses antibody-based profiling by immunofluorescence confocal microscopy to assess main localization data of more than 12,000 genes. We used this resource to ascertain the which cellular and organelle structures the proteins in our dataset localized to. GO annotations using the QuickGO website (Binns et al., 2009) and also the PANTHER classification system (Mi et al., 2019) were also used to elucidate the cellular localizations as well as describe additional functional activities of the proteins. GO annotations are based on evidence-based statements about a particular gene and are derived from experimental, phylogenetic, and computational evidences as well as author and curatorial statements and also automatically generated annotations.

Gene enrichment in the 5TM dataset in comparison to the *Homo sapiens* transmembrane proteome was analyzed with the PANTHER Classification System (version 14.1; released 11 July 2019) (Mi et al., 2019). Fisher’s exact test was chosen for the PANTHER Overrepresentation Test, which assumes a hypergeometric distribution that is more accurate for smaller gene lists, as well as the Benjamini-Hochberg False Discovery Rate (FDR) correction (*p* < 0.05) to control the false positive rate in the statistical test results. The annotation datasets included PANTHER GO-Slim Molecular Functions, Biological Processes, and Cellular Components, as well as the GO complete sets. The reference protein list for the *Homo sapiens* membrane proteome was obtained from (Attwood et al., 2017).

### Phylogenetic Analysis

To obtain homologues of the sideroflexin family, the *Homo sapiens* SFXN1-5 protein sequences were used at NCBI BLASTp suite against specific species. Default parameters were used. The following sequences were obtained from NCBI: *M. polymorpha* (OAE21648.1); *P. patens* (XP_024391321.1); *S. moellendorffii* (XP_002960929.2); *S. punctatus* (XP_016610 670.1); *M. verticillata* (KFH62263.1); *U. maydis* (XP_011390 020.1); *C. neoformans var. grubii* (OWZ51622.1); *N. crassa* (XP_9 57698.1); *P. grisea* (XP_030977476.1); *A. fumigatus* (XP_75 3043.1); *Y. lipolytica* (XP_500957.1); *S. cerevisiae* (NP_014914.1); *S. pombe* (NP_594262.2); *D. discoideum* (XP_64000 8.1); *A. queenslandica* (XP_003383899.1, XP_003383928.1); *T. adhaerens* (XP_002116694.1, XP_002117399.1); *H. vulgaris* (XP_012564924.1, XP_002162432.1, XP_012557207.1); *L. gigantea* (XP_009052487.1, XP_009050598.1, XP_0090 64188.1); *C. gigas* (XP_011424610.1, XP_019925797.1, XP_011414570.1); *C. elegans* (NP_509949.1, NP_509341.2, NP_001309542.1); *D. pulex* (EFX72259.1, EFX71151.1); *A. pisum* (NP_001156182.1, XP_008186264.1); *A mellifera* (XP_623312.2, XP_392085.2); *D. melanogaster* (NP_649460.3, NP_649086.2); *S. purpuratus* (XP_030841332.1, XP_030842117.1, XP_0308 42111.1); *P. caudata* (XP_014665525.1, XP_014672932.1, XP_014673935.1); *D. rerio* (XP_005169684.1, XP_005169 528.1, NP_001074133.1, NP_001070130.1, XP_021336710.1); *X. tropicalis* (NP_001016244.1, XP_012821950.1, NP_001135 699.1, XP_017951466.1, NP_001004915.1); *G. gallus* (XP_02 5010610.1, XP_421731.1, XP_015144246.1, XP_001234861.3, XP_420891.4); *M. musculus* (NP_081600.1, NP_444426.3, NP_001349310.1, NP_444428.3, XP_006506879.1); and *H. sapiens* (NP_001309906, XP_024303560.1, NP_112233.2, XP_005269582.1, NP_653180.1). *S. fallax* (Sphfalx0019s0101.1) was obtained from the JGI Phytozome resource (Nordberg et al., 2014). A multiple sequence alignment was obtained using MAFFT with the E-INS-i iterative refinement method and JTT substitution matrix. The alignment was manually curated. Phylogenetic topology was constructed using Bayesian inference with MrBayes version 3.2.7a to generate tree support using posterior probabilities (Huelsenbeck and Ronquist, 2001). The number of substitution sites was 6, the mixed amino acid model was used, and the simulation was run for 1,000,000 generations, with sampling every 100 and burnin fraction was 0.25. The PROTGAMMAAUTO model in RAxML was used with 500 bootstrap replicates.

All analysis and classifications were performed using local Python and Perl scripts and SQL databases (sqlite3). Adobe Illustrator CS6 was used for the figures.

## Data Availability Statement

The datasets presented in this study can be found in online repositories. The names of the repository/repositories and accession number(s) can be found in the article/Supplementary Material.

## Author Contributions

MA contributed to conceiving the project, created the dataset, analyzed the data, and drafted the manuscript. HS contributed to conceiving the project and editing the manuscript. Both authors contributed to the article and approved the submitted version.

## Conflict of Interest

The authors declare that the research was conducted in the absence of any commercial or financial relationships that could be construed as a potential conflict of interest.

## Footnotes

1 http://www.proteinatlas.org

## Supplementary Material

Supplementary Figure 1 Overview of methods and the bioinformatic tools used in this analysis.

Supplementary Table 1 Table of identified protein-disease associations.

Supplementary Data Sheet List of accession numbers used in this analysis.
