# Supplementary material for: Characterization of Five Transmembrane Proteins: With Focus on the Tweety, Sideroflexin, and YIP1 Domain Families
Source: Front Cell Dev Biol. 2021 Jul 19;9:708754. doi: 10.3389/fcell.2021.708754 (PMC8327215; doi:10.3389/fcell.2021.708754)
Supplement: Supplementary Figure 1 — Overview of methods and the bioinformatic tools used in this analysis. [file Image_1.pdf]

Supplementary Figure 1

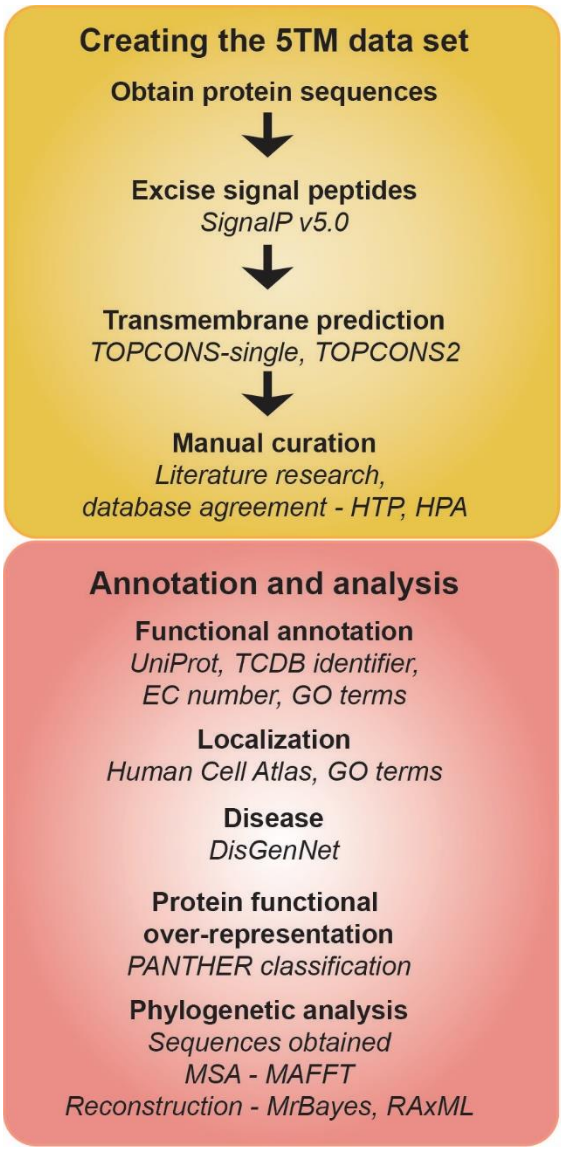

Supplementary Figure 1. Methods diagram. Creating the 5TM data set included obtaining the protein sequences, preparing the sequences by using SignalP v5.0 to identify the signal peptides and then excise them from the sequences. Transmembrane prediction software applications, including TOPCONS-single and TOPCONS2, were then utilized on the mature sequences. Manual curation followed and included researching literature and comparing results with other databases. The annotation and analysis of the 5TM data set then followed using the identified methods and databases. Abbreviations: HTP, Human Transmembrane Proteome; HPA, Human Protein Atlas; TCDB, Transporter Classification Database; EC, Enzyme Commision; GO, Gene Ontology; PANTHER, Protein Analysis Through Evolutionary Relationships; MSA, Multiple Sequence Alignnment.
