# Supplementary material for: Characterization of Five Transmembrane Proteins: With Focus on the Tweety, Sideroflexin, and YIP1 Domain Families
Source: Front Cell Dev Biol. 2021 Jul 19;9:708754. doi: 10.3389/fcell.2021.708754 (PMC8327215; doi:10.3389/fcell.2021.708754)
Supplement: Supplementary Table 1 — Table of identified protein-disease associations. [file Table_1.pdf]

Supplementary Table 1. Gene-disease associations.

**Gene**	**Potential involvement in diseases**	**Reference**
SFXN1	May be involved in cancer cell growth; sub-expressed in Alzheimer's, depression and dementia	(Minjarez et al., 2016; Fang et al., 2018; Kory et al., 2018)
SFXN2	May be involved in cancer cell growth	(Kory et al., 2018)
SFXN3	May be involved in cancer cell growth	(Labuschagne et al., 2014; Kory et al., 2018)
SFXN4	Mitochondrial disorders, complex I deficiency, macrocytic anemia, optic nerve hypoplasia	(Sofou et al., 2019)
TTYH1, TTYH2, TTYH3	Potentially associated with cerebral edema following excessive oxidative stress, ischemia, traumatic brain injury, and glioma	(Han et al., 2019)
TTYH2	Renal cell carcinoma, colon cancer	(Rae et al., 2001; Toiyama et al., 2007)
YIPF1A, YIPF1B	Possibly linked to motor neuron degeneration in amyotrophic lateral scleroses type 8	(Kuijpers et al., 2013)
YIPF4	Potential interaction with human papillomaviruses	(Shaik et al., 2019)
YIPF6	Prostate cancer	(Djusberg et al., 2017)
ARV1	Epilepsy, intellectual disabilities	(Piñero et al., 2020)
EBP	Cataracts, musculoskeletal diseases, intellectual disabilities	(Piñero et al., 2020)
DAGLA	Neurodegenerative disorders	(Piñero et al., 2020)
DUOXA2	Thyroid disorders	(Piñero et al., 2020)
PROM1	Macular degeneration, glioblastoma neoplasms	(Piñero et al., 2020)
OXA1L	Mitochondrial diseases	(Piñero et al., 2020)

References

Djusberg, E., Jernberg, E., Thysell, E., Golovleva, I., Lundberg, P., Crnalic, S., et al. (2017). High levels of the AR-V7 Splice Variant and Co-Amplification of the Golgi Protein

Coding YIPF6 in AR Amplified Prostate Cancer Bone Metastases. *The Prostate* 77, 625–638. doi:10.1002/pros.23307.

Fang, Y.-Y., Zeng, P., Qu, N., Ning, L.-N., Chu, J., Zhang, T., et al. (2018). Evidence of altered depression and dementia-related proteins in the brains of young rats after ovariectomy. *J. Neurochem.* 146, 703–721. doi:10.1111/jnc.14537.

Han, Y.-E., Kwon, J., Won, J., An, H., Jang, M. W., Woo, J., et al. (2019). Tweety-homolog (Ttyh) Family Encodes the Pore-forming Subunits of the Swelling-dependent Volume-regulated Anion Channel (VRACswell) in the Brain. *Exp. Neurobiol.* 28, 183–215. doi:10.5607/en.2019.28.2.183.

Kory, N., Wyant, G. A., Prakash, G., Bos, J. uit de, Bottanelli, F., Pacold, M. E., et al. (2018). SFXN1 is a mitochondrial serine transporter required for one-carbon metabolism. *Science* 362. doi:10.1126/science.aat9528.

Kuijpers, M., Yu, K. L., Teuling, E., Akhmanova, A., Jaarsma, D., and Hoogenraad, C. C. (2013). The ALS8 protein VAPB interacts with the ER–Golgi recycling protein YIF1A and regulates membrane delivery into dendrites. *EMBO J.* 32, 2056–2072. doi:10.1038/emboj.2013.131.

Labuschagne, C. F., van den Broek, N. J. F., Mackay, G. M., Vousden, K. H., and Maddocks, O. D. K. (2014). Serine, but Not Glycine, Supports One-Carbon Metabolism and Proliferation of Cancer Cells. *Cell Rep.* 7, 1248–1258. doi:10.1016/j.celrep.2014.04.045.

Minjarez, B., Calderón-González, K. G., Rustarazo, Ma. L. V., Herrera-Aguirre, M. E., Labra-Barrios, M. L., Rincon-Limas, D. E., et al. (2016). Identification of proteins that are differentially expressed in brains with Alzheimer's disease using iTRAQ labeling and tandem mass spectrometry. *J. Proteomics* 139, 103–121. doi:10.1016/j.jprot.2016.03.022.

Piñero, J., Ramírez-Anguita, J. M., Saüch-Pitarch, J., Ronzano, F., Centeno, E., Sanz, F., et al. (2020). The DisGeNET knowledge platform for disease genomics: 2019 update. *Nucleic Acids Res.* 48, D845–D855. doi:10.1093/nar/gkz1021.

Rae, F. K., Hooper, J. D., Eyre, H. J., Sutherland, G. R., Nicol, D. L., and Clements, J. A. (2001). TTYH2, a human homologue of the Drosophila melanogaster gene tweety, is located on 17q24 and upregulated in renal cell carcinoma. *Genomics* 77, 200–207. doi:10.1006/geno.2001.6629.

Shaik, S., Pandey, H., Thirumalasetti, S. K., and Nakamura, N. (2019). Characteristics and Functions of the Yip1 Domain Family (YIPF), Multi-Span Transmembrane Proteins Mainly Localized to the Golgi Apparatus. *Front. Cell Dev. Biol.* 7. doi:10.3389/fcell.2019.00130.

Sofou, K., Hedberg-Oldfors, C., Kollberg, G., Thomsen, C., Wiksell, Å., Oldfors, A., et al. (2019). Prenatal onset of mitochondrial disease is associated with sideroflexin 4 deficiency. *Mitochondrion* 47, 76–81. doi:10.1016/j.mito.2019.04.012.

Toiyama, Y., Mizoguchi, A., Kimura, K., Hiro, J., Inoue, Y., Tutumi, T., et al. (2007). TTYH2, a human homologue of the Drosophila melanogaster gene tweety, is up-

regulated in colon carcinoma and involved in cell proliferation and cell aggregation. *World J. Gastroenterol. WJG* 13, 2717–2721. doi:10.3748/wjg.v13.i19.2717.
