# Supplementary material for: Characterization of Five Transmembrane Proteins: With Focus on the Tweety, Sideroflexin, and YIP1 Domain Families
Source: Front Cell Dev Biol. 2021 Jul 19;9:708754. doi: 10.3389/fcell.2021.708754 (PMC8327215; doi:10.3389/fcell.2021.708754)
Supplement: Supplementary Data Sheet — List of accession numbers used in this analysis. [file Data_Sheet_1.docx]

Data obtained and utilized in this study can be found in online repositories. Protein sequence data were obtained from NCBI including the following species with accession/version number(s):

*Marchantia polymorpha subsp. ruderalis*

OAE21648.1; <https://www.ncbi.nlm.nih.gov/protein/OAE21648.1>

*Physcomitrium patens*

XP_024391321.1; <https://www.ncbi.nlm.nih.gov/protein/XP_024391321.1>

*Selaginella moellendorffii*

XP_002960929.2; <https://www.ncbi.nlm.nih.gov/protein/XP_002960929.2>

*Spizellomyces punctatus DAOM BR117*

XP_016610670.1; <https://www.ncbi.nlm.nih.gov/protein/XP_016610670.1>

*Podila verticillata NRRL 6337*

KFH62263.1; <https://www.ncbi.nlm.nih.gov/protein/KFH62263.1>

*Ustilago maydis 521*

XP_011390020.1; <https://www.ncbi.nlm.nih.gov/protein/XP_011390020.1>

*Cryptococcus neoformans var. grubii 125.91*

OWZ51622.1; <https://www.ncbi.nlm.nih.gov/protein/OWZ51622.1>

*Neurospora crassa OR74A*

XP_957698.1; <https://www.ncbi.nlm.nih.gov/protein/XP_957698.1>

*Pyricularia grisea*

XP_030977476.1; <https://www.ncbi.nlm.nih.gov/protein/XP_030977476.1>

*Aspergillus fumigatus Af293*

XP_753043.1; <https://www.ncbi.nlm.nih.gov/protein/XP_753043.1>

*Yarrowia lipolytica CLIB122*

XP_500957.1; <https://www.ncbi.nlm.nih.gov/protein/XP_500957.1>

*Saccharomyces cerevisiae S288C*

NP_014914.1; <https://www.ncbi.nlm.nih.gov/protein/NP_014914.1>

*Schizosaccharomyces pombe*

NP_594262.2; <https://www.ncbi.nlm.nih.gov/protein/NP_594262.2>

*Dictyostelium discoideum AX4*

XP_640008.1; <https://www.ncbi.nlm.nih.gov/protein/XP_640008.1>

*Amphimedon queenslandica*

XP_003383899.1; <https://www.ncbi.nlm.nih.gov/protein/XP_003383899.1>

XP_003383928.1; <https://www.ncbi.nlm.nih.gov/protein/XP_003383928.1>

*Trichoplax adhaerens*

XP_002116694.1; <https://www.ncbi.nlm.nih.gov/protein/XP_002116694.1>

XP_002117399.1; <https://www.ncbi.nlm.nih.gov/protein/XP_002117399.1>

*Hydra vulgaris*

XP_012564924.1; <https://www.ncbi.nlm.nih.gov/protein/XP_012564924.1>

XP_002162432.1; <https://www.ncbi.nlm.nih.gov/protein/XP_002162432.1>

XP_012557207.1; <https://www.ncbi.nlm.nih.gov/protein/XP_012557207.1>

*Lottia gigantea*

XP_009052487.1; <https://www.ncbi.nlm.nih.gov/protein/XP_009052487.1>

XP_009050598.1; <https://www.ncbi.nlm.nih.gov/protein/XP_009050598.1>

XP_009064188.1; <https://www.ncbi.nlm.nih.gov/protein/XP_009064188.1>

*Crassostrea gigas*

XP_011424610.1; <https://www.ncbi.nlm.nih.gov/protein/XP_011424610.1>

XP_019925797.1; <https://www.ncbi.nlm.nih.gov/protein/XP_019925797.1>

XP_011414570.1; <https://www.ncbi.nlm.nih.gov/protein/XP_011414570.1>

*Caenorhabditis elegans*

NP_509949.1; <https://www.ncbi.nlm.nih.gov/protein/NP_509949.1>

NP_509341.2; <https://www.ncbi.nlm.nih.gov/protein/NP_509341.2>

NP_001309542.1; <https://www.ncbi.nlm.nih.gov/protein/NP_001309542.1>

*Daphnia pulex*

EFX72259.1; <https://www.ncbi.nlm.nih.gov/protein/EFX72259.1>

EFX71151.1; <https://www.ncbi.nlm.nih.gov/protein/EFX71151.1>

*Acyrthosiphon pisum*

NP_001156182.1; <https://www.ncbi.nlm.nih.gov/protein/NP_001156182.1>

XP_008186264.1; <https://www.ncbi.nlm.nih.gov/protein/XP_008186264.1>

*Apis mellifera*

XP_623312.2; <https://www.ncbi.nlm.nih.gov/protein/XP_623312.2>

XP_392085.2; <https://www.ncbi.nlm.nih.gov/protein/XP_392085.2>

*Drosophila melanogaster*

NP_649460.3; <https://www.ncbi.nlm.nih.gov/protein/NP_649460.3>

NP_649086.2; <https://www.ncbi.nlm.nih.gov/protein/NP_649086.2>

*Strongylocentrotus purpuratus*

XP_030841332.1; <https://www.ncbi.nlm.nih.gov/protein/XP_030841332.1>

XP_030842117.1; <https://www.ncbi.nlm.nih.gov/protein/XP_030842117.1>

XP_030842111.1; <https://www.ncbi.nlm.nih.gov/protein/XP_030842111.1>

*Priapulus caudatus*

XP_014665525.1; <https://www.ncbi.nlm.nih.gov/protein/XP_014665525.1>

XP_014672932.1; <https://www.ncbi.nlm.nih.gov/protein/XP_014672932.1>

XP_014673935.1; <https://www.ncbi.nlm.nih.gov/protein/XP_014673935.1>

*Danio rerio*

XP_005169684.1; <https://www.ncbi.nlm.nih.gov/protein/XP_005169684.1>

XP_005169528.1; <https://www.ncbi.nlm.nih.gov/protein/XP_005169528.1>

NP_001074133.1; <https://www.ncbi.nlm.nih.gov/protein/NP_001074133.1>

NP_001070130.1; <https://www.ncbi.nlm.nih.gov/protein/NP_001070130.1>

XP_021336710.1; <https://www.ncbi.nlm.nih.gov/protein/XP_021336710.1>

*Xenopus tropicalis*

NP_001016244.1; <https://www.ncbi.nlm.nih.gov/protein/NP_001016244.1>

XP_012821950.1; <https://www.ncbi.nlm.nih.gov/protein/XP_012821950.1>

NP_001135699.1; <https://www.ncbi.nlm.nih.gov/protein/NP_001135699.1>

XP_017951466.1; <https://www.ncbi.nlm.nih.gov/protein/XP_017951466.1>

NP_001004915.1; <https://www.ncbi.nlm.nih.gov/protein/NP_001004915.1>

*Gallus gallus*

XP_025010610.1; <https://www.ncbi.nlm.nih.gov/protein/XP_025010610.1>

XP_421731.1; <https://www.ncbi.nlm.nih.gov/protein/XP_421731.1>

XP_015144246.1; <https://www.ncbi.nlm.nih.gov/protein/XP_015144246.1>

XP_001234861.3; <https://www.ncbi.nlm.nih.gov/protein/XP_001234861.3>

XP_420891.4; <https://www.ncbi.nlm.nih.gov/protein/XP_420891.4>

*Mus musculus*

NP_081600.1; <https://www.ncbi.nlm.nih.gov/protein/NP_081600.1>

NP_444426.3; <https://www.ncbi.nlm.nih.gov/protein/NP_444426.3>

NP_001349310.1; <https://www.ncbi.nlm.nih.gov/protein/NP_001349310.1>

NP_444428.3; <https://www.ncbi.nlm.nih.gov/protein/NP_444428.3>

XP_006506879.1; <https://www.ncbi.nlm.nih.gov/protein/XP_006506879.1>

*Homo sapiens*

NP_001309906; <https://www.ncbi.nlm.nih.gov/protein/NP_001309906>

XP_024303560.1; <https://www.ncbi.nlm.nih.gov/protein/XP_024303560.1>

NP_112233.2; <https://www.ncbi.nlm.nih.gov/protein/NP_112233.2>

XP_005269582.1; <https://www.ncbi.nlm.nih.gov/protein/XP_005269582.1>

NP_653180.1; <https://www.ncbi.nlm.nih.gov/protein/NP_653180.1>
